# Leveraging osteoclast genetic regulatory data to identify genes with a role in osteoarthritis

**DOI:** 10.1093/genetics/iyad150

**Published:** 2023-08-14

**Authors:** Benjamin H Mullin, Kun Zhu, Suzanne J Brown, Shelby Mullin, Frank Dudbridge, Nathan J Pavlos, J Brent Richards, Elin Grundberg, Jordana T Bell, Eleftheria Zeggini, John P Walsh, Jiake Xu, Scott G Wilson

**Affiliations:** Department of Endocrinology and Diabetes, Sir Charles Gairdner Hospital, Nedlands, WA 6009, Australia; School of Biomedical Sciences, University of Western Australia, Crawley, WA 6009, Australia; Department of Endocrinology and Diabetes, Sir Charles Gairdner Hospital, Nedlands, WA 6009, Australia; Medical School, University of Western Australia, Crawley, WA 6009, Australia; Department of Endocrinology and Diabetes, Sir Charles Gairdner Hospital, Nedlands, WA 6009, Australia; Department of Endocrinology and Diabetes, Sir Charles Gairdner Hospital, Nedlands, WA 6009, Australia; School of Biomedical Sciences, University of Western Australia, Crawley, WA 6009, Australia; Department of Population Health Sciences, University of Leicester, Leicester LE1 7RH, UK; School of Biomedical Sciences, University of Western Australia, Crawley, WA 6009, Australia; Department of Twin Research and Genetic Epidemiology, King's College London, London SE1 7EH, UK; Department of Medicine, Human Genetics, Epidemiology, and Biostatistics, Jewish General Hospital, McGill University, Montreal H3A 0G4, Canada; Genomic Medicine Center, Children’s Mercy Kansas City, Children’s Mercy Research Institute, Kansas City, MO 64108, USA; Department of Twin Research and Genetic Epidemiology, King's College London, London SE1 7EH, UK; Helmholtz Zentrum München—German Research Center for Environmental Health, Institute of Translational Genomics, Neuherberg 85764, Germany; TUM School of Medicine, Technical University of Munich (TUM) and Klinikum Rechts der Isar, Munich 81675, Germany; Department of Endocrinology and Diabetes, Sir Charles Gairdner Hospital, Nedlands, WA 6009, Australia; Medical School, University of Western Australia, Crawley, WA 6009, Australia; School of Biomedical Sciences, University of Western Australia, Crawley, WA 6009, Australia; Chinese Academy of Sciences, Shenzhen Institute of Advanced Technology, Shenzhen 518055, China; Department of Endocrinology and Diabetes, Sir Charles Gairdner Hospital, Nedlands, WA 6009, Australia; School of Biomedical Sciences, University of Western Australia, Crawley, WA 6009, Australia; Department of Twin Research and Genetic Epidemiology, King's College London, London SE1 7EH, UK

**Keywords:** osteoclast, gene expression, expression quantitative trait locus, genome-wide association study, colocalization, osteoarthritis

## Abstract

There has been a growing interest in the role of the subchondral bone and its resident osteoclasts in the progression of osteoarthritis (OA). A recent genome-wide association study (GWAS) identified 100 independent association signals for OA traits. Most of these signals are led by noncoding variants, suggesting that genetic regulatory effects may drive many of the associations. We have generated a unique human osteoclast-like cell-specific expression quantitative trait locus (eQTL) resource for studying the genetics of bone disease. Considering the potential role of osteoclasts in the pathogenesis of OA, we performed an integrative analysis of this dataset with the recently published OA GWAS results. Summary data-based Mendelian randomization (SMR) and colocalization analyses identified 38 genes with a potential role in OA, including some that have been implicated in Mendelian diseases with joint/skeletal abnormalities, such as *BICRA*, *EIF6*, *CHST3*, and *FBN2*. Several OA GWAS signals demonstrated colocalization with more than one eQTL peak, including at 19q13.32 (hip OA with *BCAM*, *PRKD2*, and *BICRA* eQTL). We also identified a number of eQTL signals colocalizing with more than one OA trait, including *FAM53A*, *GCAT*, *HMGN1*, *MGAT4A*, *RRP7BP*, and *TRIOBP*. An SMR analysis identified 3 loci with evidence of pleiotropic effects on OA-risk and gene expression: *LINC01481*, *CPNE1*, and *EIF6*. Both *CPNE1* and *EIF6* are located at 20q11.22, a locus harboring 2 other strong OA candidate genes, *GDF5* and *UQCC1*, suggesting the presence of an OA-risk gene cluster. In summary, we have used our osteoclast-specific eQTL dataset to identify genes potentially involved with the pathogenesis of OA.

## Introduction

Osteoarthritis (OA) is a degenerative articular disease and the most prevalent form of arthritis (Lawrence *et al.* 2008; Zhang *et al.* 2016). It is a leading cause of disability globally, affecting 40% of individuals over 70 years of age (Vos *et al.* 2012), and its prevalence is increasing with population aging and rising rates of obesity (Cross *et al.* 2014). OA can affect any of the synovial joints in the body, but it is most commonly observed in the knees, hips, hands, and spine. It is a whole-joint disease, characterized by changes in the hyaline articular cartilage, subchondral bone, periarticular muscles, joint capsule, synovial membrane, and ligaments (Brandt *et al.* 2006), and can lead to the pathological stage of arthrofibrosis (Usher *et al.* 2019). In recent years, there has been growing interest in the role of the subchondral bone and its resident osteoclasts in the progression of OA. Increased turnover and subsequent sclerosis of the subchondral bone are common features of the disease, along with the development of subchondral bone marrow lesions and vascular invasion into the cartilage (Henrotin *et al.* 2012). Studies in equine carpal bone suggest that mechanical overloading of the joint facilitates the release of osteoclastogenic cytokines, resulting in increased osteoclast recruitment to the subchondral plate contributing to subchondral bone loss and the formation of microcracks in the calcified cartilage (Bertuglia *et al.* 2016). Other studies have demonstrated that osteoclasts are capable of resorbing both calcified and noncalcified cartilages independently of acidification within the joint (Löfvall *et al.* 2018) and that pharmacological suppression of bone resorption can improve cartilage health (Karsdal *et al.* 2008). Subchondral osteoclasts have also been shown to secrete factors that promote sensory innervation of the subchondral bone, leading to OA-related pain (Zhu *et al.* 2019).

A number of modifiable risk factors for OA have been identified, including previous joint injury and high body mass index (Cooper *et al.* 2000). In addition to these, the disease has a significant genetic contribution, with twin studies suggesting the heritability of radiographic knee and hip OA to be around 39% (Spector *et al.* 1996) and 60% (MacGregor *et al.* 2000), respectively. Genome-wide association studies (GWASs) have successfully identified a number of genetic loci associated with the disease (Tachmazidou *et al.* 2019; Boer *et al.* 2021) as well as related traits such as hip shape (Baird *et al.* 2019). The largest of these performed to date was published by Boer *et al.* (2021) and represents a meta-analysis of 9 populations including the UK Biobank, for a total sample size of 177,517 OA cases and 649,173 controls. This study identified 100 independent genome-wide significant association signals for the disease, including 52 located in genetic loci not previously associated with OA. As is the case with most complex diseases, the vast majority of these signals are led by noncoding variants located in intergenic or intronic DNA, suggesting that underlying regulatory effects on gene expression may be driving the associations. However, for many of these genetic loci, the effector genes responsible for the association have yet to be determined.

Expression quantitative trait locus (eQTL) studies have proved useful for the identification of GWAS effector genes through characterization of associations between the genetic sequence and gene expression. A recent study used eQTL data from primary cartilage and synovium samples obtained from OA patients to identify a number of genes potentially involved in the disease (Steinberg *et al.* 2021). However, few eQTL studies have been performed in other cell types relevant to OA. We have previously generated a unique human osteoclast-specific eQTL resource for study of the genetics of bone disease and have successfully used this to identify a number of genetic regulatory effects for osteoporosis and Paget's disease risk variants (Mullin *et al.* 2018, 2019, 2020). Considering the potential role of osteoclasts and subchondral bone remodeling in the pathogenesis of OA, we decided to integrate the data from our osteoclast eQTL resource with the OA GWAS summary results published by Boer *et al.* (2021) in multiomics analyses to check for evidence of osteoclast-specific genetic regulatory effects that may contribute to OA pathobiology.

## Materials and methods

### Patient recruitment and osteoclastogenesis

A detailed description of the methods used to generate the osteoclast eQTL dataset has been published previously (Mullin *et al.* 2018). Briefly, 158 females aged 18–70 undergoing dual-energy X-ray absorptiometry bone mineral density (BMD) scanning (Hologic, Bedford, MA, USA) were recruited from the Bone Density Unit at Sir Charles Gairdner Hospital in Western Australia. Any subjects suffering from medical conditions or taking medications likely to affect osteoclast function or differentiation were excluded from the study. All patients provided written informed consent, and the study was approved by the Sir Charles Gairdner and Osborne Park Health Care Group Human Research Ethics Committee. Peripheral blood mononuclear cells (PBMCs) were isolated from lithium heparin blood samples obtained from each patient using density gradient centrifugation and cultured to differentiate into osteoclast-like cells in vitro (Mullin *et al.* 2014). Isolated PBMCs were cultured in triplicate, initially for 2 days in complete *α*-minimum essential medium (MEM) supplemented with 25 ng/mL macrophage colony stimulating factor (M-CSF) and then for an additional 12 days in complete *α*-MEM supplemented with 25 ng/mL M-CSF and 100 ng/mL receptor activator of nuclear factor kappa-B ligand to stimulate osteoclastogenesis. Gene expression profiling and staining of the cells for tartrate resistant acid phosphatase were performed to confirm the osteoclast phenotype.

### Extraction of nucleic acids

Genomic DNA was extracted from ethylenediaminetetraacetic acid (EDTA) blood samples obtained from each patient using the QIAamp DNA Blood Mini Kit (QIAGEN) according to the manufacturer's instructions. Total RNA and genomic DNA were harvested from the cultured osteoclast-like cells using the AllPrep DNA/RNA Mini Kit (QIAGEN) according to the manufacturer's instructions. Once cell lysis had been performed, the lysate from each set of triplicate cultures was combined into a single aliquot for DNA/RNA purification.

### Genotyping and imputation

Each genomic DNA sample extracted from EDTA whole blood was subjected to genome-wide array genotyping using the Illumina Infinium OmniExpress-24 BeadChip array. Preimputation quality control (QC) criteria applied included removal of samples with a call rate <90% or variants that were monomorphic, had a minor allele frequency (MAF) <5%, had Hardy–Weinberg equilibrium *P* < 5 × 10^−8^, or had a call rate <90%. Genotype imputation was performed on 572,898 variants using the Sanger Imputation Service employing the Haplotype Reference Consortium release 1.1 reference panel (McCarthy *et al.* 2016). Any imputed variants with an IMPUTE2 info score <0.4 were removed from the genotype dataset. Relatedness testing and principal component analysis was performed on the preimputation genotype dataset using Plink v1.9 (Chang *et al.* 2015), with 10 principal components retained for use as covariates in the eQTL analysis.

### Gene expression data processing

Gene expression quantitation was performed on the osteoclast RNA samples using 50 bp single-end RNA-Seq on an Illumina HiSeq 2500. Raw read counts were generated for each gene (GENCODE v25), with genes not achieving >1 count per million (CPM) expression in at least 10 samples removed from the dataset. The normalization of the RNA-Seq data was performed using the trimmed mean of M-values (TMM) method, with correction for total read count performed by converting the data to CPM using the edgeR package in the R statistical computing environment (Robinson *et al.* 2010).

### Identification of eQTL associations

The FastQTL software (Ongen *et al.* 2016) was used to identify eQTL associations for the TMM-normalized CPM gene expression values, with quantile normalization implemented. The association analysis was corrected for the covariates: RNA-Seq batch, patient age, and 10 genomic principal components. Each variant was tested for association with the expression level of any gene with a transcription start site (TSS) located within 2 Mb on either side of the variant. Only variants with a MAF ≥ 5% were included in the analysis.

### Colocalization analysis

Colocalization of OA GWAS and osteoclast eQTL association signals was assessed using the coloc2 package in R (Dobbyn *et al.* 2018), which is an updated version of the original coloc package (Giambartolomei *et al.* 2014) that includes additional features from gwas-pw (Pickrell *et al.* 2016). This software generates posterior probabilities using a Bayesian method for 5 different hypotheses describing the sharing of causal variants between the 2 genetic association datasets. A posterior probability of ≥80% for hypothesis H_4_ (single variant affecting both traits) is considered to be strong Bayesian evidence for colocalization, while a posterior probability ≥50% for hypothesis H_4_ is considered moderate evidence for colocalization.

### Summary data-based Mendelian randomization analysis

An integrative analysis of the osteoclast eQTL (±1 Mb) and OA GWAS datasets was performed using summary data-based Mendelian randomization (SMR) software (Zhu *et al.* 2016) to identify potential pleiotropic effects on osteoclast gene expression and OA. This package applies the principles of Mendelian randomization (Smith and Ebrahim 2003) to examine whether the effect of a genetic variant on a trait is mediated through gene expression. The software performs an SMR test whereby the top eQTL variant for each gene is used as an instrumental variable to test for association between the expression of that gene and the GWAS trait. The heterogeneity in dependent instruments (HEIDI) test is then used to check for heterogeneity in the association profiles of nearby coinherited markers. The presence of heterogeneity in the association profiles of the 2 datasets, as indicated by a significant HEIDI test (*P*_HEIDI_ < 0.05), is indicative of a linkage scenario (2 different causal variants), which is of less biological relevance than pleiotropy (single variant affecting both traits). The 1,000 Genomes Project phase 3 dataset (Auton *et al.* 2015) was used as the linkage disequilibrium reference for this analysis, and only genes with an eQTL association significant at *P <* 5 × 10^−8^ were included. For the SMR test, correction for multiple testing was performed using the Benjamini–Hochberg method (Benjamini and Hochberg 1995).

## Results

The demographics of the osteoclast eQTL cohort are presented in Supplementary Table 1. Using the lowest T score among the 3 sites measured (total hip, femoral neck, and lumbar spine), the proportion of participants with low bone density (T score between −1 and −2.5) and osteoporosis (T score ≤ −2.5) was found to be 51.2 and 17.1%, respectively. All subjects had self-reported European ancestry, none were found to be closely related (all PI_HAT <0.06), and principal components analysis confirmed that there were no ethnic outliers in the population. The eQTL analysis included 5,373,348 imputed variants with a MAF ≥ 5% and 15,688 gene transcripts that were found to be expressed in the osteoclast-like cells after the QC criteria were applied. For the multiomics analyses, we used the summary GWAS results for 4 of the best-powered OA traits studied by Boer *et al.* (2021). These include all OA (i.e. OA at any site, including the hip, knee, hand, finger, thumb, and spine) (*N* = 177,517 cases, 649,173 controls), knee OA (*N* = 62,497 cases, 333,557 controls), hip OA (*N* = 36,445 cases, 316,943 controls), and knee and/or hip OA (*N* = 89,741 cases, 400,604 controls). Over 97% of the variants in the eQTL dataset were also present in the OA GWAS datasets.

### Colocalization analysis

Colocalization analysis was performed using coloc2 (Dobbyn *et al.* 2018) to identify colocalized association signals in the OA GWAS results and the osteoclast eQTL dataset. The presence of colocalized association signals in GWAS and eQTL datasets indicates a shared genetic effect on the 2 traits, suggesting that the GWAS association may be mediated by regulatory effects on the eQTL gene. For all OA, knee OA, hip OA, and knee and/or hip OA, evidence of colocalization with eQTL association signals (posterior probability ≥50%) was seen for 7, 8, 14, and 13 loci, respectively (Table 1; Supplementary Table 2). Of these, 0, 1, 5, and 4 demonstrated strong evidence of colocalization (posterior probability ≥80%), respectively.

**Table 1.** Colocalized loci identified in the OA GWAS and osteoclast eQTL datasets.

Trait	Gene	Top GWAS hit	*P*	Top eQTL hit	*P*	Best causal	PP H_4_
All OA	*ALKBH5*	rs854772	1.8E−7	rs2605132	9.4E−7	rs860567	0.67
	*TSEN15*	rs10911472	1.2E−6	rs17572231	5.4E−7	rs11582961	0.67
	*HMGN1*	rs9981884	1.3E−7	rs8128901	9.5E−11	rs8128901	0.66
	*GMPPB*	rs62262106	2.1E−8	rs1005678	1.2E−5	rs62263602	0.60
	*ZFP1*	rs8057775	2.0E−5	rs12716781	3.1E−13	rs8057775	0.53
	*RAI1*	rs854772	1.8E−7	rs854781	4.7E−4	rs854772	0.53
	*RRP7BP*	rs137123	1.8E−5	rs137133	2.4E−6	rs137127	0.50
Knee OA	***CMB9-55F22.1***	**rs76560824**	**6.0E−7**	**rs4963153**	**1.4E−12**	**rs4963153**	**0**.**83**
	*DHX32*	rs61870942	2.4E−5	rs11244733	1.9E−12	rs61870942	0.76
	*HMGN1*	rs9981884	2.4E−6	rs8128901	9.5E−11	rs8128901	0.74
	*MGAT4A*	rs6715321	7.6E−8	rs6715321	3.5E−4	rs1839666	0.72
	*CEP57L1P1*	rs9416019	2.0E−7	rs6480615	7.3E−4	rs9415063	0.67
	*CAMK2G*	rs9416019	2.0E−7	rs7897548	9.6E−4	rs7897548	0.59
	*LIN7C*	rs1517572	5.2E−6	rs1038659	3.1E−7	rs1038659	0.56
	*FUT11*	rs9416019	2.0E−7	rs7897548	1.9E−3	rs9415063	0.52
Hip OA	***GCAT***	**rs12160491**	**4.4E−10**	**rs12160750**	**6.5E−6**	**rs12160491**	**0**.**90**
	***FAM53A***	**rs798756**	**2.2E−9**	**rs798727**	**1.1E−6**	**rs798756**	**0**.**88**
	***TRIOBP***	**rs12160491**	**4.4E−10**	**rs2413485**	**2.3E−5**	**rs12160491**	**0**.**87**
	***PLEC***	**rs7464572**	**2.1E−6**	**rs11784833**	**1.9E−5**	**rs7464572**	**0**.**84**
	***ZNF618***	**rs1330349**	**6.9E−12**	**rs1755637**	**3.1E−5**	**rs1330351**	**0**.**80**
	*PRKD2*	rs2238689	9.6E−7	rs2238689	7.3E−5	rs2238689	0.75
	*BCAM*	rs2238689	9.6E−7	rs2238689	7.7E-5	rs2238689	0.71
	*BICRA*	rs2238689	9.6E−7	rs2238689	1.1E−4	rs2238689	0.67
	*MVB12B*	rs62578126	4.3E−11	rs13298228	4.1E−4	rs62578126	0.67
	*ZBED4*	rs9616205	3.6E−6	rs112796939	3.4E−6	rs112796939	0.65
	*GNL3*	rs2268023	1.6E−13	rs6778329	5.3E−5	rs678	0.57
	*FBN2*	rs17677724	3.5E−9	rs6870746	2.5E−14	rs10040416	0.55
	*CHST3*	rs3740129	1.8E−9	rs59514134	1.5E−4	rs1245535	0.54
	*PIM3*	rs9616205	3.6E−6	rs73179172	6.8E−5	rs62233158	0.51
Knee and/or hip OA	***HMGN1***	**rs9981884**	**3.3E−8**	**rs8128901**	**9.5E−11**	**rs8128901**	**0**.**94**
	***GCAT***	**rs6000899**	**4.0E−7**	**rs12160750**	**6.5E−6**	**rs12160750**	**0**.**93**
	***TRIOBP***	**rs6000899**	**4.0E−7**	**rs2413485**	**2.3E−5**	**rs12160491**	**0**.**89**
	***MGAT4A***	**rs6715321**	**3.7E−7**	**rs6715321**	**3.5E−4**	**rs6761390**	**0**.**84**
	*RP11-436D10.3*	rs2236295	2.9E−6	rs10995311	8.6E−13	rs2236295	0.78
	*PSME4*	rs57154773	2.4E−7	rs117296869	2.4E−4	rs57154773	0.74
	*PCDH1*	rs6580203	4.6E−7	rs6580203	3.6E−4	rs6580203	0.62
	*REEP5*	rs2439591	7.0E−6	rs2545165	1.5E−6	rs501250	0.61
	*FAM53A*	rs59163323	2.9E−12	rs798727	1.1E−6	rs59163323	0.58
	*LYG1*	rs6715321	3.7E−7	rs2048949	7.0E−5	rs2309576	0.57
	*PTCH1*	rs2282040	3.2E−5	rs28377268	9.3E−9	rs28418339	0.55
	*TGFA*	rs3755381	4.2E−13	rs1833350	1.5E−4	rs3755381	0.53
	*RRP7BP*	rs137132	3.9E−5	rs137133	2.4E−6	rs137127	0.51

PP H_4_, posterior probability of hypothesis H4 (single variant affecting both traits). Best causal: variant with the highest posterior probability to be the true causal variant. The results for loci with strong evidence for colocalization (posterior probability ≥80%) are shown in bold.

Several GWAS association signals demonstrated colocalization with more than one eQTL peak, such as those located at 2q11.2 (knee and/or hip OA with *LYG1* and *MGAT4A*), 10q22.2 (knee OA with *CEP57L1P1*, *FUT11*, and *CAMK2G*), 17p11.2 (all OA with *RAI1* and *ALKBH5*), 19q13.32 (hip OA with *BCAM*, *PRKD2*, and *BICRA*), 22q13.1 (hip OA and knee and/or hip OA with *TRIOBP* and *GCAT*), and 22q13.33 (hip OA with *ZBED4* and *PIM3*). This phenomenon is suggestive of either an eQTL influencing the expression of more than one gene within the locus as well as OA risk or multiple eQTLs within a locus independently colocalizing with OA GWAS association signals.

**Fig. 1.**
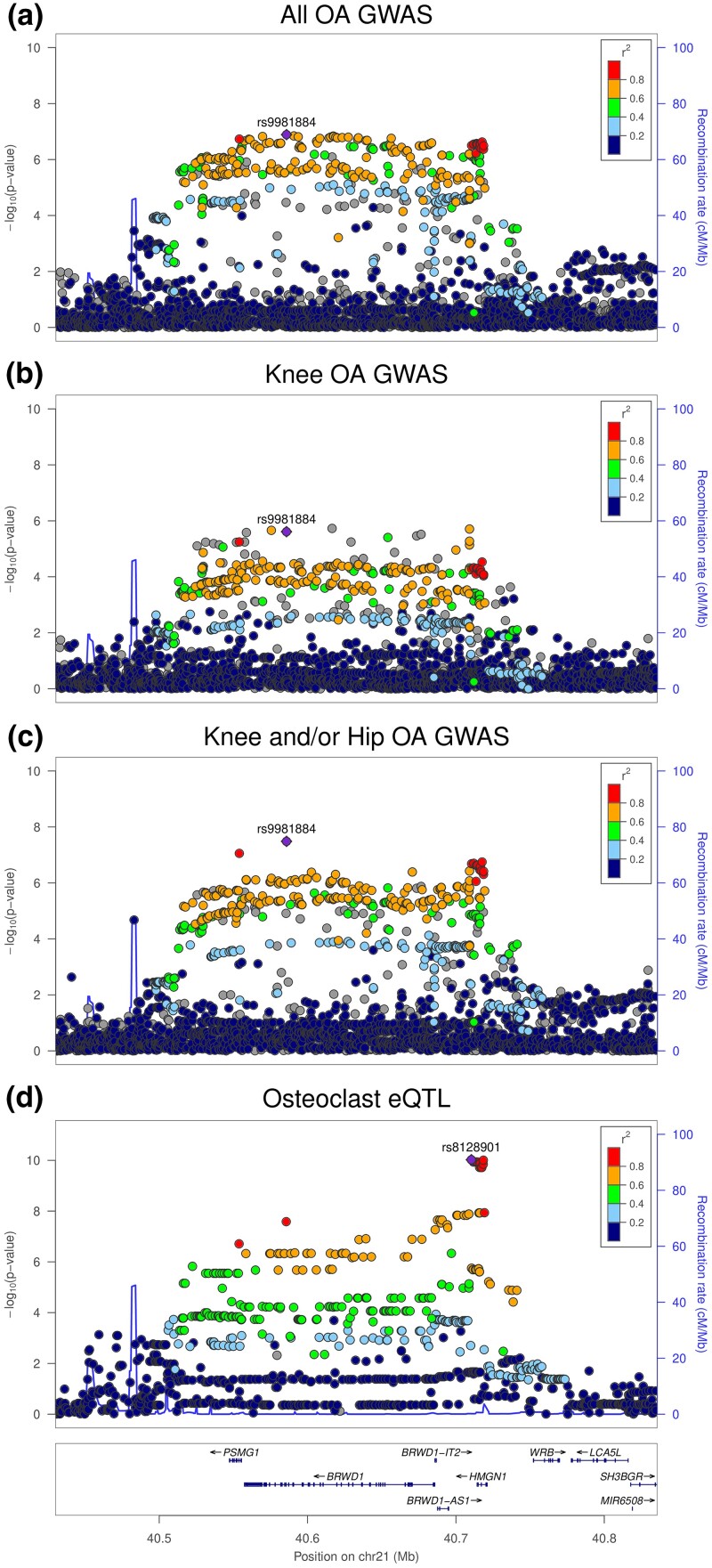
Association plots generated using the summary results for a) all OA GWAS, b) knee OA GWAS, c) knee and/or hip OA GWAS, and d) osteoclast eQTL results for the *HMGN1* gene. Strong evidence of colocalization was observed for each of these OA GWAS association signals with the *HMGN1* osteoclast eQTL results (66–94% posterior probability). The GWAS variant rs9981884 is in strong linkage disequilibrium with the top eQTL variant for *HMGN1*, rs8128901 [*r*^2^ = 0.84 in Europeans (Machiela and Chanock 2015)]. Genetic variants are depicted according to chromosomal location on the *x*-axis and associated *P*-value (−log10) on the *y*-axis.

A number of eQTL signals also demonstrated colocalization with GWAS peaks for multiple OA traits, including *FAM53A* (hip OA and knee and/or hip OA), *GCAT* (hip OA and knee and/or hip OA), *HMGN1* (all OA, knee OA, and knee and/or hip OA) (Fig. 1), *MGAT4A* (knee OA and knee and/or hip OA), *RRP7BP* (all OA and knee and/or hip OA), and *TRIOBP* (hip OA and knee and/or hip OA).

### SMR analysis

We performed an integrative analysis of the osteoclast eQTL and OA GWAS datasets using the SMR software (Zhu *et al.* 2016), which uses the principles of Mendelian randomization to test for pleiotropic associations between gene expression levels and a trait of interest. A total of 1,073 genes were identified with GWAS results present and an eQTL association significant at *P* < 5 × 10^−8^. After correction for multiple testing, we identified 13 genes as significantly associated with an OA trait: 3 for all OA, 5 for knee OA, and 5 for knee and/or hip OA (Table 2). No significant associations were identified in the SMR test for hip OA.

**Table 2.** Significant associations identified in the SMR analysis.

Trait	Gene	eQTL	*P*_GWAS_	*P*_eQTL_	*P*_SMR_	*Q*_SMR_	*β*_SMR_	*P*_HEIDI_
All OA	*HMGN1*	rs8128901	3.2E−7	9.5E−11	6.6E−5	0.048	−0.03	5.3E−8
	***LINC01481***	**rs117979485**	**5.7E−6**	**1.1E−12**	**1.2E−4**	**0**.**048**	**0**.**03**	**0.45**
	*UHRF1BP1*	rs9380455	3.3E−5	4.3E−24	1.4E−4	0.048	−0.02	7.3E−7
Knee OA	*EXOSC6*	rs2070203	3.7E−6	3.8E−24	2.4E−5	0.013	−0.04	0.02
	*UHRF1BP1*	rs9380455	3.9E−6	4.3E−24	2.5E−5	0.013	−0.03	2.6E−7
	*CMB9-55F22.1*	rs4963153	3.8E−6	1.4E−12	1.1E−4	0.036	0.05	1.4E−5
	***CPNE1***	**rs6060535**	**4.0E−5**	**2.2E−21**	**1.6E−4**	**0**.**036**	**0**.**03**	**0.09**
	***EIF6***	**rs111916968**	**7.0E−7**	**8.4E−9**	**1.7E−4**	**0**.**036**	**−0**.**07**	**0.97**
Knee and/or hip OA	*HMGN1*	rs8128901	2.1E−7	9.5E−11	5.1E−5	0.033	−0.04	3.8E−8
	***EIF6***	**rs111916968**	**2.2E−8**	**8.4E−9**	**6.1E−5**	**0**.**033**	**−0**.**06**	**0.92**
	*LYNX1*	rs13276915	1.7E−5	3.9E−17	1.3E−4	0.046	−0.03	4.9E−4
	*RP11-436D10.3*	rs10995311	1.4E−5	8.6E−13	2.0E−4	0.046	−0.03	1.6E−4
	***LINC01481***	**rs117979485**	**1.6E−5**	**1.1E−12**	**2.1E−4**	**0**.**046**	**0**.**03**	**0.75**

*P*_GWAS_, *P*-value for the eQTL variant in the GWAS dataset; *P*_eQTL_, *P*-value for the eQTL variant in the eQTL dataset; *P*_SMR_, *P*-value for the SMR test; *Q*_SMR_, Benjamini–Hochberg adjusted *P*-value for the SMR test; *β*_SMR_, beta value derived from the SMR analysis; *P*_HEIDI_, *P*-value from the HEIDI test. All loci are significantly associated with the OA trait in the SMR test at the 5% false discovery rate. The loci demonstrating evidence of a shared causal variant, as indicated by *P*_HEIDI_  *≥* 0.05, are highlighted in bold.

Of the 3 significant associations identified in the SMR test for all OA, the gene *LINC01481* demonstrated a nonsignificant result in the HEIDI test, suggesting the presence of a shared causal variant in the GWAS and eQTL datasets. The expression of *LINC01481* was found to be positively associated with the risk of developing OA at any site (*β*_SMR_ = 0.03, Supplementary Fig. 1).

**Fig. 2.**
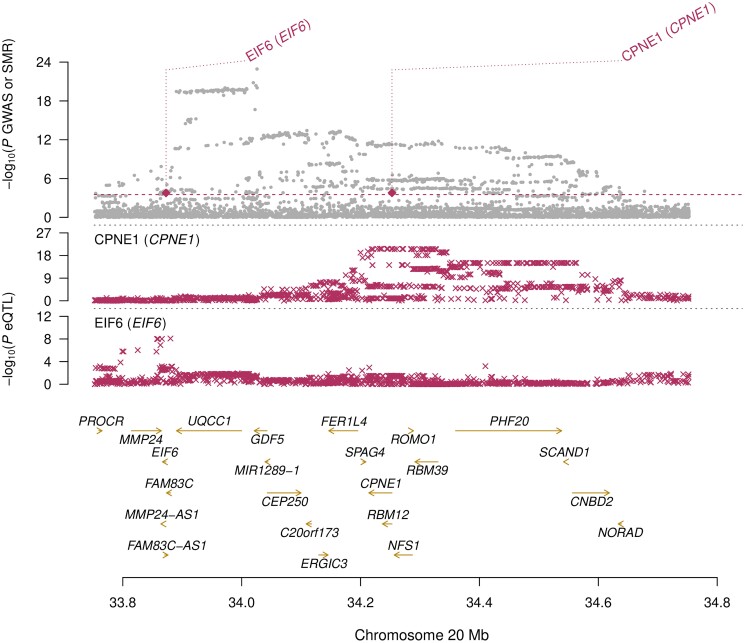
SMR plot of the *CPNE1*/*EIF6* locus. The upper panel depicts the knee OA GWAS *P*-values (gray points), the middle panels depict the *CPNE1* and *EIF6* eQTL *P*-values, and the lower panel depicts the gene locations on chromosome 20. The shaded diamonds in the upper panel represent the SMR test *P*-values for the *CPNE1* (*P* = 1.61 × 10^−4^) and *EIF6* (*P* = 1.68 × 10^−4^) genes, with the significance threshold indicated by the dashed line.

For the knee OA trait, 2 genes demonstrated nonsignificant HEIDI test results: *CPNE1* and *EIF6*. The expression of *CPNE1* was found to be positively associated with the risk of developing knee OA (*β*_SMR_ = 0.03), whereas the expression of *EIF6* was found to be negatively associated with the risk of developing knee OA (*β*_SMR_ = −0.07). Both *CPNE1* and *EIF6* are located within the same region on chromosome 20 (Fig. 2).

Two of the 5 significant genes identified in the SMR test for knee and/or hip OA demonstrated likely shared causal variants in the GWAS and eQTL datasets: *EIF6* and *LINC01481*. The expression of *EIF6* was again found to be negatively associated with OA risk (*β*_SMR_ = −0.06, Supplementary Fig. 2), whereas the expression of *LINC01481* was positively associated with knee and/or hip OA (*β*_SMR_ = 0.03, Supplementary Fig. 3).

## Discussion

In this multiomics study, we have capitalized on our osteoclast-like cell-specific eQTL dataset to identify several potential OA GWAS effector genes. The *HMGN1* gene presented with strong evidence for colocalization of OA GWAS and osteoclast eQTL association signals across 3 different OA traits and in fact demonstrated the strongest probability of colocalization out of all loci analyzed (94% posterior probability of colocalization with knee and/or hip OA). This indicates the presence of a shared causal variant influencing the expression of *HMGN1* and susceptibility to OA, suggesting that the genetic association with OA traits at this locus may be mediated through regulatory effects on *HMGN1*. However, it should be noted that this finding was not replicated in the SMR analysis, in which the *HMGN1* gene consistently produced significant results for the HEIDI test. A possible explanation for this is the presence of more than one causal variant within the locus, which could create heterogeneity in the 2 datasets. The *HMGN1* gene encodes the high mobility group nucleosome binding domain 1 protein, which has a role in modulating chromatin structure and has also been shown to act as an alarmin, inducing antigen-specific immune responses (Yang *et al.* 2012). The results from our analysis suggest that the greater expression of *HMGN1* may lead to an increased risk of developing OA. It has previously been shown that the expression of *HMGN1* is significantly upregulated in OA compared with normal cartilage (Taniguchi *et al.* 2009). In addition, a recent study performed RNA-Seq in 65 articular cartilage samples collected from OA patients and demonstrated an allelic expression imbalance for a variant located in the 3′ untranslated region of *HMGN1* (rs3167757) (Coutinho de Almeida *et al.* 2022), which is in strong linkage disequilibrium with the lead OA GWAS variant identified by Boer *et al.* at the *HMGN1* locus (rs9981884). The results from that study suggest that the less common *T* allele at rs3167757, which is linked with the less common *A* allele at rs9981884, is associated with reduced expression of *HMGN1* in cartilage. The results from this previous study and our own both suggest that the OA risk-increasing *G* allele at rs9981884 is associated with increased expression of *HMGN1*.

An interesting variant highlighted by this study is rs2238689, which was associated with hip OA in the study by Boer *et al.* (2021), albeit at only at the genome-wide suggestive level (*P =* 9.6 × 10^−7^). This variant was the most significantly associated eQTL hit for 3 genes in our osteoclast gene expression dataset: *BCAM*, *PRKD2*, and *BICRA* (also known as *GLTSCR1*). The hip OA risk-increasing *T* allele at rs2238689 was associated with reduced expression of *BCAM* and increased expression of *PRKD2* and *BICRA*. All 3 of these genes are located >500 kb from rs2238689, and there are no other variants in strong linkage disequilibrium (*r*^2^ > 0.5) with this SNP in Europeans (Machiela and Chanock 2015), suggesting that it could be located within a long-range control element. This SNP is an intronic variant in the *gastric inhibitory polypeptide receptor* (*GIPR*) gene and has previously been identified as associated with several lipid ratio traits and type 2 diabetes at the genome-wide level (Mahajan *et al.* 2018; Richardson *et al.* 2022). Mutation in the *BICRA* gene has been implicated in a genetic disorder characterized by a variety of neurodevelopmental phenotypes as well as dysmorphic and orthopedic features such as short stature, scoliosis, radioulnar synostosis, joint laxicity, and hip subluxation (Barish *et al.* 2020).

Three genes were identified in the SMR analysis as having expression levels significantly associated with OA risk with evidence of pleiotropic effects: *LINC01481*, *CPNE1*, and *EIF6*. This can be interpreted as evidence for causal effects of gene expression, if it is assumed that the genetic associations with OA are mediated through regulatory effects on these genes. However, SMR (and Mendelian randomization in general) cannot distinguish this from unmediated (so-called horizontal) pleiotropic effects. The fact that the *LINC01481* and *EIF6* genes presented with significant SMR test results for more than one OA trait provides additional confidence that these genes may be involved in the pathogenesis of OA. Both the *CPNE1* and *EIF6* genes are located in the same genetic locus on chromosome 20q11.22. This is in fact the top genetic locus identified by Boer *et al.* (2021) for both the knee OA and the knee and/or hip OA traits. This presents as a fascinating genetic locus from an OA perspective, with 2 other strong OA candidate genes residing in the area, *GDF5* and *UQCC1*, both of which encode proteins involved in growth factor signaling. The product of the *CPNE1* gene is a calcium-dependent protein that may have a role in membrane trafficking. An RNA-based network analysis published by Zhang *et al.* (2019) identified the *CPNE1* gene as an OA-related core RNA molecule in human OA patients and cartilage-derived stem/progenitor cells from a mouse model of OA. The *EIF6* gene encodes an integrin-binding protein that has a role in ribosomal biogenesis (Sanvito *et al.* 1999). Mutation in this gene has been implicated in Shwachman–Diamond syndrome, a rare multisystem disorder characterized by a variety of symptoms including failure of the bone marrow and skeletal abnormalities such as osteopenia, dysplasia of the rib cage, and metaphyseal chondrodysplasia (Koh *et al.* 2020). It is possible that this genetic locus harbors an OA-risk gene cluster, which could explain the strong associations seen with OA traits.

It is worth considering our findings in the context of other studies incorporating eQTL data that have been performed in the OA field to date. As part of their OA GWAS meta-analysis, Boer *et al.* (2021) performed a colocalization analysis of their GWAS association results with eQTL data from the 48 tissues of the GTEx v7 data release (Battle *et al.* 2017). They identified several genes with strong evidence of GWAS/eQTL signal colocalization, including 9 genes that presented with at least moderate evidence of colocalization in our study: *CHST3*, *FAM53A*, *FBN2*, *GCAT*, *GNL3*, *PTCH1*, *TGFA*, *TRIOBP*, and *TSEN15*. The *CHST3* gene encodes an enzyme that catalyzes the sulfation of chondroitin-containing proteoglycans, the most abundant proteoglycan found in cartilage. Mutation in this gene has been found to cause spondyloepiphyseal dysplasia, a rare disorder with a variety of severe skeletal traits including joint dislocations and arthritic changes (Thiele *et al.* 2004). The product of the *FBN2* gene, fibrillin 2, is a structural component of the connective tissue microfibrils. Mutation in this gene has been implicated in congenital contractural arachnodactyly, a disorder characterized by joint contractures, scoliosis, and arachnodactyly (Putnam *et al.* 1995). In a separate study published by Steinberg *et al.* (2021), molecular QTL data were generated using primary cartilage and synovium samples obtained from 115 patients undergoing total joint replacement surgery. They identified strong evidence of colocalization of OA GWAS signals from a study published by Tachmazidou *et al.* (2019) with cartilage molecular QTL for 5 genes, one of which was also identified in our study, *FAM53A*. The results from our study in osteoclasts provide supporting evidence for the involvement of these genes in OA.

A limitation of this study is the entirely female makeup of the eQTL cohort. It is possible that some of the regulatory effects seen in this study relevant to OA are female specific. However, although sex-specific eQTL effects have been previously reported, they appear to be relatively uncommon, with only 369 sex-biased eQTLs detected across all of the GTEx tissues (Oliva *et al.* 2020). Another potential limitation is the relatively small sample size of the eQTL study cohort (*N* = 158). However, this figure is commensurate with many of the tissues in the GTEx Portal. Studies of genetic regulation tend to be better powered than complex disease GWAS when one tests for association with gene expression, which is directly influenced by a DNA sequence, as opposed to a disease state that may have developed over many decades and have polygenic input and substantial environmental contributions. A strength of this study is the osteoclast-specific nature of the eQTL data, a cell type not studied by the GTEx Consortium, which allows interrogation of genetic regulatory mechanisms unique to this cell type. By identifying the effector genes driving OA GWAS associations through effects in osteoclasts, we can highlight biological pathways involved in the pathophysiology of the disease, some of which may not have been previously identified. This can potentially lead to the identification of novel pharmacological targets for treatment of the disease.

In summary, we have performed integrative multiomics analyses of summary results from the largest OA GWAS published to date with our osteoclast-specific gene expression dataset using the SMR and colocalization methods. A number of potential OA GWAS effector genes have been identified, including some that have been implicated in Mendelian diseases with joint and skeletal abnormalities, such as *BICRA* (also known as *GLTSCR1*), *EIF6, CHST3*, and *FBN2*. We have also identified several loci harboring multiple colocalized eQTL/GWAS association signals, including the hip OA-risk variant rs2238689, which appears to have regulatory effects on multiple genes located in the 19q13.32 chromosomal region in this cell type. The results from this study have also highlighted a potential OA risk gene cluster on chromosome 20q11.22, consisting of *UQCC1*, *GDF5*, *CPNE1*, and *EIF6*.

## Supplementary Material

iyad150_Supplementary_Data

## Data Availability

The complete osteoclast eQTL dataset is available for download from the GEnetic Factors for OSteoporosis Consortium (GEFOS) website: http://www.gefos.org/?q=content/human-osteoclast-eqtl-2018-2020. The OA GWAS summary results are publicly available for download from the Musculoskeletal Knowledge Portal: https://msk.hugeamp.org/datasets.html. Supplemental material available at GENETICS online.
