# Supplementary material for: Leveraging osteoclast genetic regulatory data to identify genes with a role in osteoarthritis
Source: Genetics. 2023 Aug 14;225(2):iyad150. doi: 10.1093/genetics/iyad150 (PMC10550309; doi:10.1093/genetics/iyad150)
Supplement: iyad150_Supplementary_Data [file iyad150_supplementary_data.zip › Supplemental_Figure_Legends_GENETICS-2023-306301.docx]

**Leveraging osteoclast genetic regulatory data to identify genes with a role in osteoarthritis**

Benjamin H Mullin^1,2^, Kun Zhu^1,3^, Suzanne J Brown^1^, Shelby Mullin^1,2^, Frank Dudbridge^4^, Nathan J Pavlos^2^, J Brent Richards^5,6^, Elin Grundberg^7^, Jordana T Bell^5^, Eleftheria Zeggini^8,9^, John P Walsh^1,3^, Jiake Xu^2,10^, Scott G Wilson^1,2,5^

^1^Department of Endocrinology & Diabetes, Sir Charles Gairdner Hospital, Nedlands, WA, 6009, Australia

^2^School of Biomedical Sciences, University of Western Australia, Crawley, WA, 6009, Australia

^3^Medical School, University of Western Australia, Crawley, WA, 6009, Australia

^4^Department of Population Health Sciences, University of Leicester, Leicester, LE1 7RH, UK

^5^Department of Twin Research & Genetic Epidemiology, King’s College London, London, SE1 7EH, UK

^6^Departments of Medicine, Human Genetics, Epidemiology, and Biostatistics, Jewish General Hospital, McGill University, Montreal, H3A 0G4, Canada

^7^Genomic Medicine Center, Children's Mercy Kansas City, Children's Mercy Research Institute, Kansas City, MO, 64108, USA

^8^Institute of Translational Genomics, Helmholtz Zentrum München – German Research Center for Environmental Health, Neuherberg, 85764, Germany

^9^Technical University of Munich (TUM) and Klinikum Rechts der Isar, TUM School of Medicine, Munich, 81675, Germany

^10^Shenzhen Institute of Advanced Technology, Chinese Academy of Sciences, Shenzhen, 518055, China

Corresponding author

Benjamin H. Mullin

School of Biomedical Sciences

The University of Western Australia

Crawley, Western Australia 6009

T: +61 8 6457 2466

E-mail: [Benjamin.Mullin@uwa.edu.au](mailto:Benjamin.Mullin@uwa.edu.au)

ORCID ID: 0000-0003-0743-770X

**Supplementary Figure S1.** SMR plot of the *LINC01481* locus. The upper panel depicts the all OA GWAS *P*-values (grey points), the middle panel the *LINC01481* eQTL *P*-values and the lower panel the gene locations on chromosome 12. The red diamond in the upper panel represents the SMR test *P*-value for the *LINC01481* gene (*P = 1.23×10^-4^*), with the significance threshold indicated by the dashed red line.

**Supplementary Figure S2.** SMR plot of the *EIF6* locus. The upper panel depicts the kneeand/or hip OA GWAS *P*-values (grey points), the middle panel the *EIF6* eQTL *P*-values and the lower panel the gene locations on chromosome 20. The red diamond in the upper panel represents the SMR test *P*-value for the *EIF6* gene (*P = 6.1×10^-5^*), with the significance threshold indicated by the dashed red line.

**Supplementary Figure S3.** SMR plot of the *LINC01481* locus. The upper panel depicts the knee and/or hip OA GWAS *P*-values (grey points), the middle panel the *LINC01481* eQTL *P*-values and the lower panel the gene locations on chromosome 12. The red diamond in the upper panel represents the SMR test *P*-value for the *LINC01481* gene (*P = 2.14×10^-4^*), with the significance threshold indicated by the dashed red line.
