# Supplementary material for: Leveraging osteoclast genetic regulatory data to identify genes with a role in osteoarthritis
Source: Genetics. 2023 Aug 14;225(2):iyad150. doi: 10.1093/genetics/iyad150 (PMC10550309; doi:10.1093/genetics/iyad150)
Supplement: iyad150_Supplementary_Data [file iyad150_supplementary_data.zip › Supplemental_Tables_GENETICS-2023-306301.docx]

**Leveraging osteoclast genetic regulatory data to identify genes with a role in osteoarthritis**

Benjamin H Mullin^1,2^, Kun Zhu^1,3^, Suzanne J Brown^1^, Shelby Mullin^1,2^, Frank Dudbridge^4^, Nathan J Pavlos^2^, J Brent Richards^5,6^, Elin Grundberg^7^, Jordana T Bell^5^, Eleftheria Zeggini^8,9^, John P Walsh^1,3^, Jiake Xu^2,10^, Scott G Wilson^1,2,5^

^1^Department of Endocrinology & Diabetes, Sir Charles Gairdner Hospital, Nedlands, WA, 6009, Australia

^2^School of Biomedical Sciences, University of Western Australia, Crawley, WA, 6009, Australia

^3^Medical School, University of Western Australia, Crawley, WA, 6009, Australia

^4^Department of Population Health Sciences, University of Leicester, Leicester, LE1 7RH, UK

^5^Department of Twin Research & Genetic Epidemiology, King’s College London, London, SE1 7EH, UK

^6^Departments of Medicine, Human Genetics, Epidemiology, and Biostatistics, Jewish General Hospital, McGill University, Montreal, H3A 0G4, Canada

^7^Genomic Medicine Center, Children's Mercy Kansas City, Children's Mercy Research Institute, Kansas City, MO, 64108, USA

^8^Institute of Translational Genomics, Helmholtz Zentrum München – German Research Center for Environmental Health, Neuherberg, 85764, Germany

^9^Technical University of Munich (TUM) and Klinikum Rechts der Isar, TUM School of Medicine, Munich, 81675, Germany

^10^Shenzhen Institute of Advanced Technology, Chinese Academy of Sciences, Shenzhen, 518055, China

Corresponding author

Benjamin H. Mullin

School of Biomedical Sciences

The University of Western Australia

Crawley, Western Australia 6009

T: +61 8 6457 2466

E-mail: [Benjamin.Mullin@uwa.edu.au](mailto:Benjamin.Mullin@uwa.edu.au)

ORCID ID: 0000-0003-0743-770X

**Supplementary Table S1.** Osteoclast eQTL cohort demographics

| **Variable** | **Mean (SD)** |
| --- | --- |
| Subjects (n) | 158 |
| Age (years) | 57.2 (9.7) |
| Age (range) | 30.5 – 69.8 |
| Height (cm) | 162.7 (6.2) |
| Weight (kg) | 67.7 (13.0) |
| BMI (kg/m^2^) | 25.6 (5.1) |
| Spine BMD (g/cm^2^) | 0.94 (0.15) |
| Spine BMD T-score | -0.96 (1.34) |
| Total hip BMD (g/cm^2^) | 0.84 (0.12) |
| Total hip BMD T-score | -0.80 (0.99) |
| Femoral neck BMD (g/cm^2^) | 0.72 (0.11) |
| Femoral neck BMD T-score | -1.17 (0.96) |

SD: standard deviation, BMD: bone mineral density.

**Supplementary Table S2.** Effects on gene expression and OA risk for the best causal variants identified in the co-localisation analysis

| **Variant** | **Location** | **EA** | **OA** | **EAF** | **Gene** | **β_eQTL_ (95%CI)** | ***P*_eQTL_** | **OA site** | **β_GWAS_** | ***P*_GWAS_** |
| --- | --- | --- | --- | --- | --- | --- | --- | --- | --- | --- |
| rs11582961 | 1:184023059 | T | C | 0.11 | *TSEN15* | 0.89 (0.53,1.25) | 9.3E-7 | All | 0.04 | 2.8E-6 |
| rs57154773 | 2:54179018 | T | C | 0.09 | *PSME4* | -0.68 (-1.08,-0.28) | 9.3E-4 | Knee/hip | -0.05 | 2.4E-7 |
| rs3755381 | 2:70718695 | C | T | 0.49 | *TGFA* | -0.29 (-0.5,-0.08) | 8.2E-3 | Knee/hip | -0.04 | 4.2E-13 |
| rs2309576 | 2:99972355 | T | C | 0.41 | *LYG1* | 0.37 (0.16,0.58) | 5.7E-4 | Knee/hip | 0.03 | 5.3E-7 |
| rs1839666 | 2:100102942 | A | G | 0.42 | *MGAT4A* | -0.43 (-0.67,-0.19) | 3.5E-4 | Knee | 0.04 | 1.1E-7 |
| rs6761390 | 2:100106797 | A | C | 0.42 | *MGAT4A* | -0.43 (-0.67,-0.19) | 3.5E-4 | Knee/hip | 0.03 | 4.9E-7 |
| rs62263602 | 3:50152491 | T | C | 0.26 | *GMPPB* | 0.54 (0.26,0.82) | 2.0E-4 | All | 0.03 | 2.3E-8 |
| rs678 | 3:52820981 | T | A | 0.34 | *GNL3* | -0.41 (-0.63,-0.19) | 2.3E-4 | Hip | 0.07 | 3.7E-13 |
| rs798756 | 4:1707447 | T | C | 0.23 | *FAM53A* | 0.71 (0.41,1.01) | 3.0E-6 | Hip | -0.07 | 2.2E-9 |
| rs59163323 | 4:1763318 | A | G | 0.23 | *FAM53A* | 0.69 (0.38,1) | 1.2E-5 | Knee/hip | -0.06 | 2.9E-12 |
| rs501250 | 5:112155793 | G | C | 0.35 | *REEP5* | 0.49 (0.29,0.69) | 1.7E-6 | Knee/hip | 0.03 | 3.7E-5 |
| rs10040416 | 5:127795824 | A | G | 0.08 | *FBN2* | 1.47 (1.09,1.85) | 3.9E-14 | Hip | -0.09 | 3.0E-8 |
| rs6580203 | 5:141257058 | C | G | 0.48 | *PCDH1* | -0.43 (-0.67,-0.19) | 3.6E-4 | Knee/hip | -0.03 | 4.6E-7 |
| rs7464572 | 8:145021167 | G | C | 0.38 | *PLEC* | -0.44 (-0.65,-0.23) | 5.5E-5 | Hip | 0.04 | 2.1E-6 |
| rs28418339 | 9:98253476 | A | G | 0.10 | *PTCH1* | 1 (0.65,1.35) | 3.1E-8 | Knee/hip | -0.04 | 3.8E-5 |
| rs1330351 | 9:117840922 | A | G | 0.50 | *ZNF618* | -0.5 (-0.74,-0.26) | 5.5E-5 | Hip | -0.06 | 1.2E-11 |
| rs62578126 | 9:129375338 | T | C | 0.36 | *MVB12B* | 0.41 (0.17,0.65) | 1.0E-3 | Hip | -0.06 | 4.3E-11 |
| rs2236295 | 10:64564892 | T | G | 0.40 | *RP11-436D10.3* | -0.81 (-1.04,-0.58) | 2.5E-12 | Knee/hip | 0.03 | 2.9E-6 |
| rs1245535 | 10:73789033 | C | G | 0.45 | *CHST3* | 0.45 (0.2,0.7) | 3.3E-4 | Hip | -0.05 | 6.3E-9 |
| rs9415063 | 10:74086652 | C | T | 0.42 | *CEP57L1P1* | -0.36 (-0.59,-0.13) | 1.9E-3 | Knee | 0.04 | 3.7E-7 |
| rs9415063 | 10:74086652 | C | T | 0.42 | *FUT11* | 0.34 (0.1,0.58) | 4.8E-3 | Knee | 0.04 | 3.7E-7 |
| rs7897548 | 10:74131542 | A | G | 0.35 | *CAMK2G* | 0.39 (0.16,0.62) | 9.6E-4 | Knee | 0.04 | 1.0E-6 |
| rs61870942 | 10:127659131 | T | C | 0.41 | *DHX32* | -0.78 (-1,-0.56) | 2.7E-12 | Knee | -0.03 | 2.5E-5 |
| rs4963153 | 11:791462 | A | G | 0.46 | *CMB9-55F22.1* | 0.69 (0.5,0.88) | 1.4E-12 | Knee | 0.03 | 3.8E-6 |
| rs1038659 | 11:27549330 | T | G | 0.21 | *LIN7C* | 0.75 (0.46,1.04) | 3.1E-7 | Knee | 0.03 | 3.0E-5 |
| rs8057775 | 16:75178714 | G | C | 0.33 | *ZFP1* | -0.87 (-1.1,-0.64) | 3.2E-13 | All | -0.02 | 2.0E-5 |
| rs860567 | 17:18043106 | A | G | 0.43 | *ALKBH5* | 0.54 (0.32,0.76) | 1.3E-6 | All | 0.02 | 9.3E-7 |
| rs854772 | 17:18055229 | G | A | 0.41 | *RAI1* | -0.35 (-0.55,-0.15) | 4.7E-4 | All | -0.02 | 1.8E-7 |
| rs2238689 | 19:46178661 | C | T | 0.35 | *PRKD2* | -0.43 (-0.64,-0.22) | 7.3E-5 | Hip | -0.04 | 9.7E-7 |
| rs2238689 | 19:46178661 | C | T | 0.35 | *BCAM* | 0.5 (0.25,0.75) | 7.8E-5 | Hip | -0.04 | 9.7E-7 |
| rs2238689 | 19:46178661 | C | T | 0.35 | *GLTSCR1* | -0.43 (-0.65,-0.21) | 1.1E-4 | Hip | -0.04 | 9.7E-7 |
| rs8128901 | 21:40710419 | G | A | 0.48 | *HMGN1* | 0.72 (0.5,0.94) | 9.5E-11 | All | 0.02 | 3.2E-7 |
| rs8128901 | 21:40710419 | G | A | 0.48 | *HMGN1* | 0.72 (0.5,0.94) | 9.5E-11 | Knee | 0.03 | 5.2E-5 |
| rs8128901 | 21:40710419 | G | A | 0.48 | *HMGN1* | 0.72 (0.5,0.94) | 9.5E-11 | Knee/hip | 0.03 | 2.1E-7 |
| rs12160491 | 22:38195796 | G | A | 0.29 | *GCAT* | 0.56 (0.31,0.81) | 1.3E-5 | Hip | 0.06 | 4.4E-10 |
| rs12160491 | 22:38195796 | G | A | 0.29 | *TRIOBP* | -0.51 (-0.75,-0.27) | 4.5E-5 | Hip | 0.06 | 4.4E-10 |
| rs12160491 | 22:38195796 | G | A | 0.29 | *TRIOBP* | -0.51 (-0.75,-0.27) | 4.5E-5 | Knee/hip | 0.03 | 4.2E-7 |
| rs12160750 | 22:38199054 | G | A | 0.30 | *GCAT* | 0.58 (0.33,0.83) | 6.6E-6 | Knee/hip | 0.03 | 4.4E-7 |
| rs137127 | 22:43019632 | A | C | 0.14 | *RRP7BP* | 0.72 (0.4,1.04) | 8.9E-6 | All | -0.03 | 2.2E-5 |
| rs137127 | 22:43019632 | A | C | 0.14 | *RRP7BP* | 0.72 (0.4,1.04) | 8.9E-6 | Knee/hip | -0.04 | 1.1E-4 |
| rs112796939 | 22:50302430 | T | C | 0.13 | *ZBED4* | 0.73 (0.42,1.04) | 3.4E-6 | Hip | -0.06 | 8.1E-6 |
| rs62233158 | 22:50305244 | C | G | 0.11 | *PIM3* | 0.64 (0.27,1.01) | 6.3E-4 | Hip | -0.07 | 5.1E-6 |

EA: effect allele, OA: other allele, EAF: effect allele frequency (derived from the osteoclast eQTL cohort), 95%CI: 95% confidence interval of the eQTL beta value, variant locations derived from human genome build GRCh37/hg19, β_GWAS_ values are relevant to the effect allele and were obtained from the study by Boer *et al.*, β_eQTL_ values are given as the normalised effect size on gene expression for the effect allele.
