# Supplementary figures and images for: Leveraging osteoclast genetic regulatory data to identify genes with a role in osteoarthritis
Source: Genetics. 2023 Aug 14;225(2):iyad150. doi: 10.1093/genetics/iyad150 (PMC10550309; doi:10.1093/genetics/iyad150)

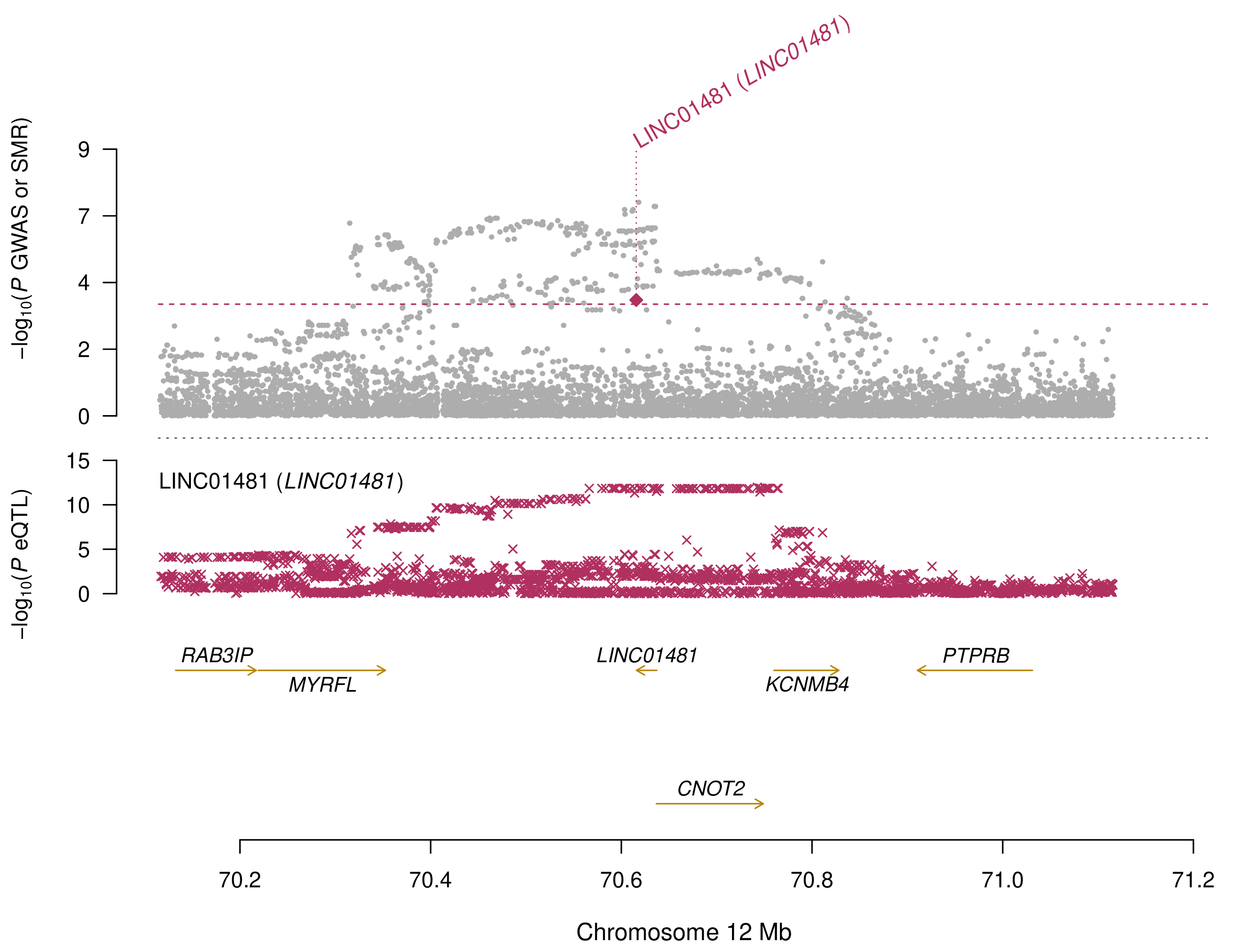

Supplement: iyad150_Supplementary_Data [file iyad150_supplementary_data.zip › Supplemental_Figure_S1_GENETICS-2023-306301.tif]

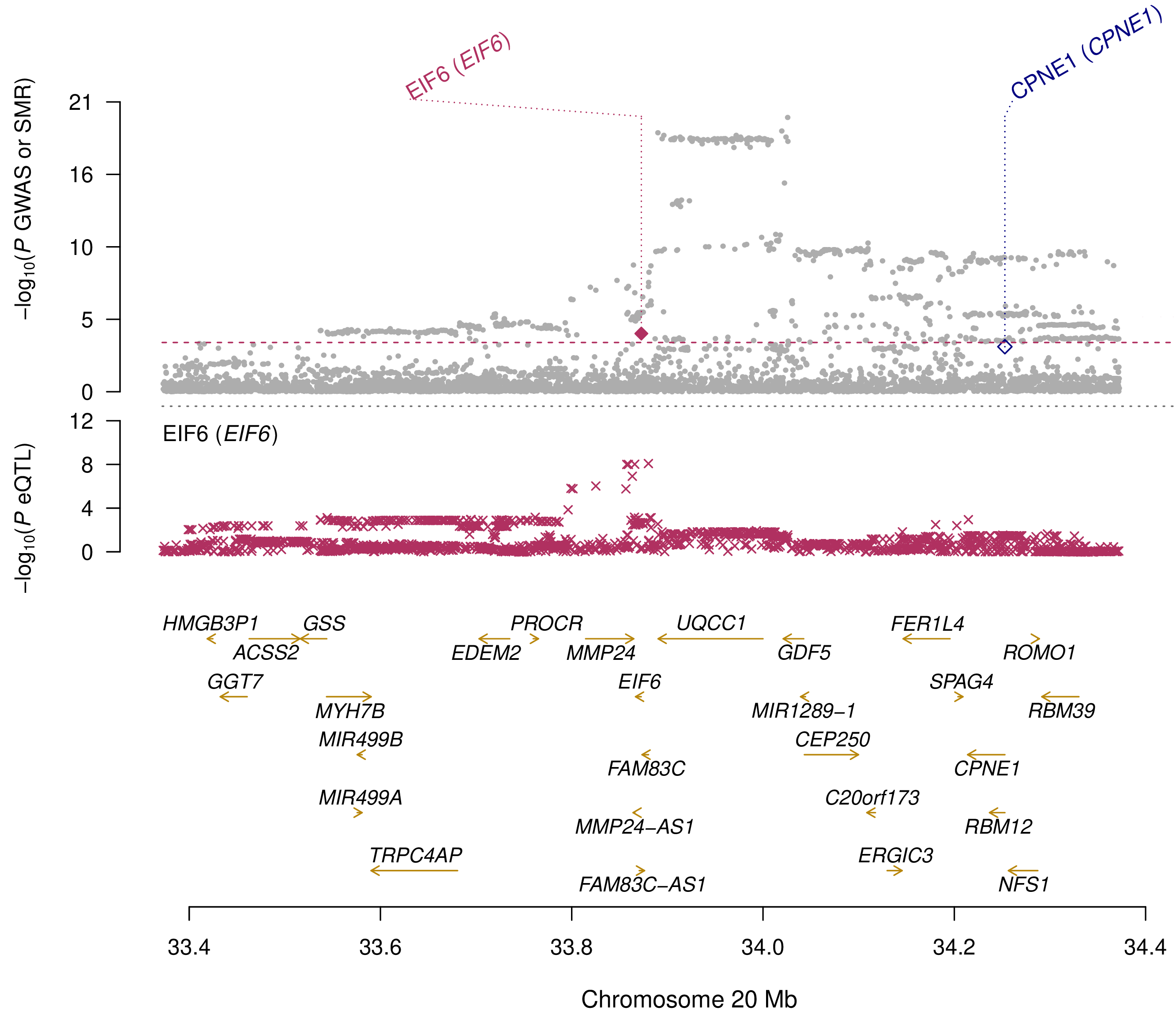

Supplement: iyad150_Supplementary_Data [file iyad150_supplementary_data.zip › Supplemental_Figure_S2_GENETICS-2023-306301.tif]

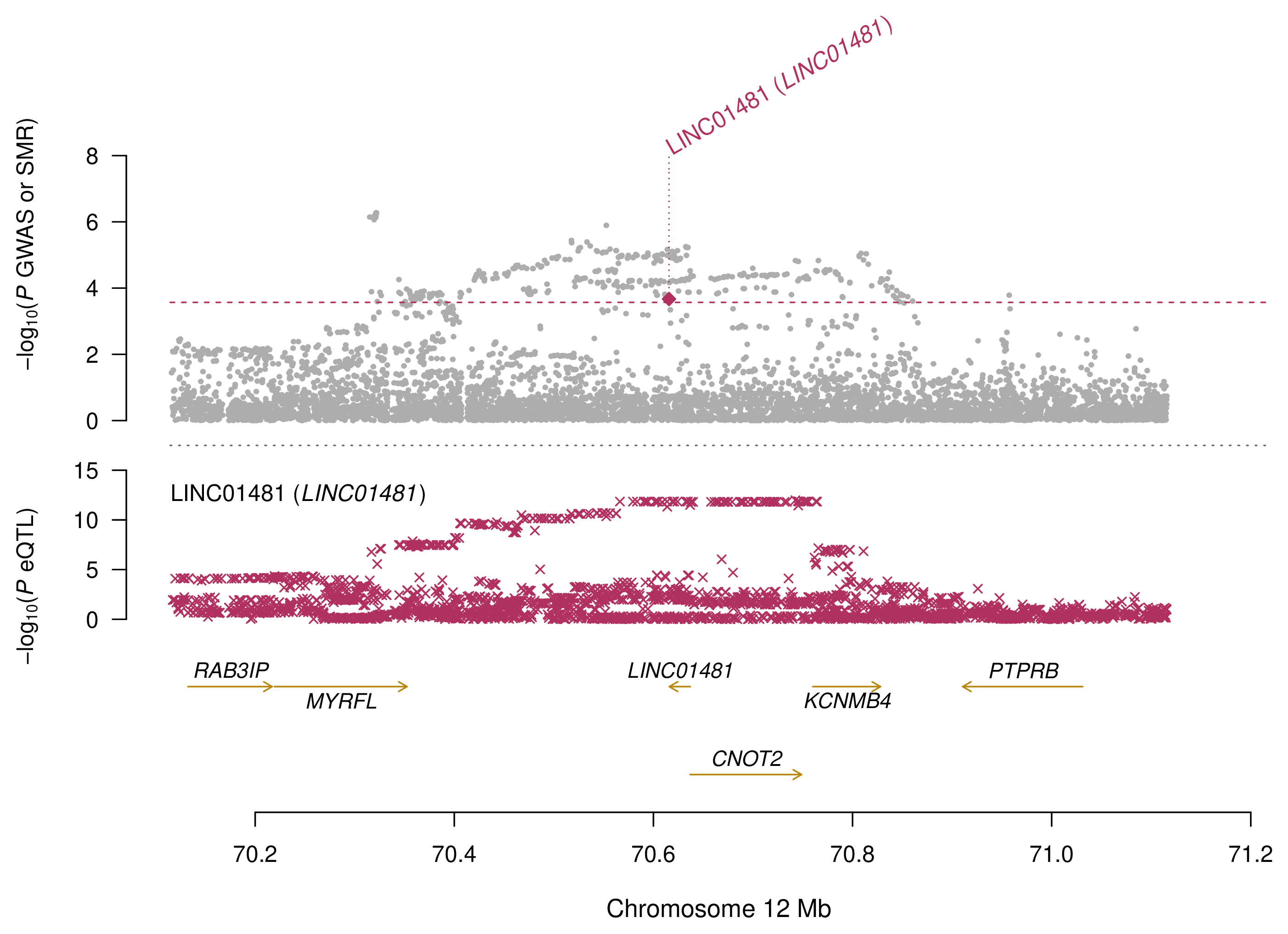

Supplement: iyad150_Supplementary_Data [file iyad150_supplementary_data.zip › Supplemental_Figure_S3_GENETICS-2023-306301.tif]
